# Radiomics in Oncological PET Imaging: A Systematic Review—Part 2, Infradiaphragmatic Cancers, Blood Malignancies, Melanoma and Musculoskeletal Cancers

**DOI:** 10.3390/diagnostics12061330

**Published:** 2022-05-27

**Authors:** David Morland, Elizabeth Katherine Anna Triumbari, Luca Boldrini, Roberto Gatta, Daniele Pizzuto, Salvatore Annunziata

**Affiliations:** 1 Unità di Medicina Nucleare, TracerGLab, Dipartimento di Diagnostica per Immagini, Radioterapia Oncologica ed Ematologia, Fondazione Policlinico Universitario A. Gemelli IRCCS, 00168 Roma, Italy; elizabethkatherineanna.triumbari@guest.policlinicogemelli.it (E.K.A.T.); danieleantonio.pizzuto@guest.policlinicogemelli.it (D.P.); salvatore.annunziata@policlinicogemelli.it (S.A.); 2 Service de Médecine Nucléaire, Institut Godinot, 51100 Reims, France; 3 Laboratoire de Biophysique, UFR de Médecine, Université de Reims Champagne-Ardenne, 51100 Reims, France; 4 CReSTIC (Centre de Recherche en Sciences et Technologies de l’Information et de la Communication), EA 3804, Université de Reims Champagne-Ardenne, 51100 Reims, France; 5 Unità di Radioterapia Oncologica, Radiomics, Dipartimento di Diagnostica per Immagini, Radioterapia Oncologica ed Ematologia, Fondazione Policlinico Universitario A. Gemelli IRCCS, 00168 Roma, Italy; luca.boldrini@policlinogemelli.it (L.B.); roberto.gatta.bs@gmail.com (R.G.); 6 Department of Clinical and Experimental Sciences, University of Brescia, 25121 Brescia, Italy; 7 Department of Oncology, Lausanne University Hospital, 1011 Lausanne, Switzerland

**Keywords:** radiomics, artificial intelligence, gastrointestinal tumors, hematological tumors, genitourinary tumors, musculoskeletal tumors, skin tumors

## Abstract

The objective of this review was to summarize published radiomics studies dealing with infradiaphragmatic cancers, blood malignancies, melanoma, and musculoskeletal cancers, and assess their quality. PubMed database was searched from January 1990 to February 2022 for articles performing radiomics on PET imaging of at least 1 specified tumor type. Exclusion criteria includd: non-oncological studies; supradiaphragmatic tumors; reviews, comments, cases reports; phantom or animal studies; technical articles without a clinically oriented question; studies including <30 patients in the training cohort. The review database contained PMID, first author, year of publication, cancer type, number of patients, study design, independent validation cohort and objective. This database was completed twice by the same person; discrepant results were resolved by a third reading of the articles. A total of 162 studies met inclusion criteria; 61 (37.7%) studies included >100 patients, 13 (8.0%) were prospective and 61 (37.7%) used an independent validation set. The most represented cancers were esophagus, lymphoma, and cervical cancer (n = 24, n = 24 and n = 19 articles, respectively). Most studies focused on 18F-FDG, and prognostic and response to treatment objectives. Although radiomics and artificial intelligence are technically challenging, new contributions and guidelines help improving research quality over the years and pave the way toward personalized medicine.

## 1. Introduction

In the recent years, radiomics has represented one of the major axes of development in medical imaging research. Similarly to all its sister disciplines (for example, genomics, proteomics, and metabolomics), this field seeks to optimize the process of discovering new disease biomarkers through a quantitative approach to medical imaging and offers an instrument to potentially build a new combination of parameters to guide patient-tailored treatment. Radiomics relies on the mathematical extraction of the spatial distribution of signal intensities and pixel interrelationships that are translated in a large number of quantitative features, the most statistically relevant parameters being then selected to deduce the purpose of the study. Thus, disease-specific textural information that are hidden to the human eye become accessible thanks to mathematical extraction. Traditional statistical approaches may have difficulties in handling such big amount of data. On the other hand, Artificial Intelligence (AI), with its ability to identify patterns within the massive dataset, has been proven very useful for this task [1].

However, though AI and radiomics are high potential-carrying techniques, they rely on rigorous processing chains and good quality training bases [2]. Yet, the quality of radiomics publications is often questioned [3], both in terms of number of patients included and lack of dedicated validation cohorts. Moreover, missing information in those studies often undermine the possibility for other researchers to replicate, and therefore externally validate, radiomics-based protocols, thus delaying the application of radiomic models in clinical practice.

As the number of articles on radiomics in oncological Positron Emission Tomography (PET) imaging exponentially increases, we here provide a systematic review, with a particular focus the quality of radiomics studies conducted on several malignancies: infradiaphragmatic cancers including gastrointestinal and genitourinary tumors; blood cancers; musculo-skeletal and skin (MSS) neoplasia.

## 2. Materials and Methods

This systematic review of published literature was performed according to the reporting standards of the PRISMA-P statement [4]. It was not registered.

### 2.1. Search Strategy, Inclusion and Exclusion Criteria

We performed a literature search in the PubMed database to identify all eligible articles using the following formula:

(“PET” OR “positron”) AND (“radiomics” OR “radiomic” OR “texture” OR “textural”)(1)

Results were admitted from 1 January 1990, up to and including 18 February 2022. Reviews were automatically identified using the article type options and removed from the extracted database.

Inclusion criteria were: (1) studies based on human data, (2) studies specifying at least one non-supradiaphragmatic tumor type, (3) studies performing radiomics on PET imaging. Exclusion criteria were: (1) studies not related to medical topics, (2) reviews, posters, editorials, comments, cases reports, (3) duplicates, (4) studies outside the oncological field or radiomics not performed on PET, (5) studies only based on phantom or animal data, (6) technical articles (optimization, robustness), without a clinically-oriented question, (7) studies including less than 30 patients in the training cohort (for studies including multiple types of cancers, each cancer type was considered separately), (8) strictly supradiaphragmatic cancers (for example, esophagus was included in this study) (9) studies not written in English, (10) full text not available (Table 1).

### 2.2. Quality Assessment

Studies were assessed for quality based on 3 items:

1. The number of patients, estimating the risk of bias and overfitting: less than 50 patients (score 0), 50 to 100 patients (score 1), more than 100 patients (score 2);
2. The retrospective (score 0) or prospective (score 2) nature of the collection of data;
3. The use of a completely independent cohort for validation: no (score 0), partition of the cohort between training and test set, excluding k-folding (score 1), external validation cohort (score 2).

A simple quality score (QS), consisting in the sum of the 3 previously stated items, was calculated. A maximum possible score of 6 meant high quality study design of the article. Mean and 95% confidence intervals (CI) of the quality scores were calculated for all of the database articles divided by year of publication.

### 2.3. Data Collection and Review

An Excel review database was generated. The following parameters were extracted from each article:

1. PMID, first author, year of publication;
2. Organ/type of cancer;
3. Quality data: number of patients, retrospective or prospective nature, validation, quality score;
4. Objective of the study.

The database was completely filled in twice by the same author, with a one-week interval between the two. Any discrepancies were corrected by a third reading.

## 3. Results

### 3.1. Discrepancies between the Two Reading Sessions

Six discrepancies between the 2 reading sessions of the database were encountered and led to a third reading: 1 was misclassified regarding cancer subtype, 5 discrepancies concerned patient number or validation cohort presence.

### 3.2. Searching Results

**Figure 1:**
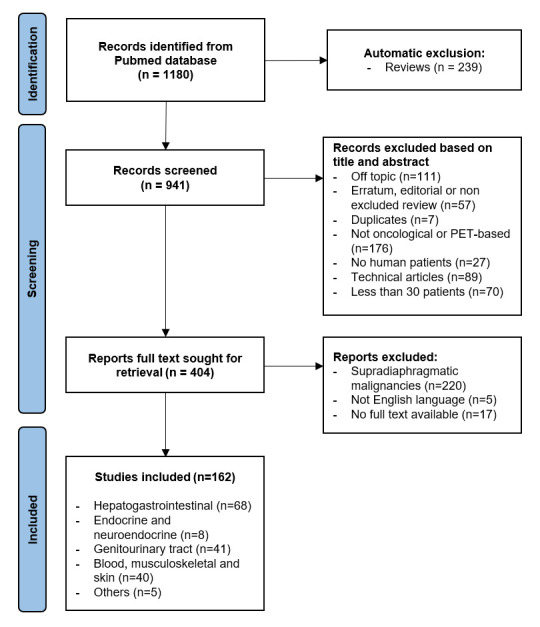
Flowchart of literature search and article selection.

A total of 1180 studies were identified in the PubMed database, 239 of which were reviews and therefore automatically excluded. Of the remaining 941 studies, 537 were excluded as 111 of them were off topic, 57 articles corresponded to undetected reviews or editorials, 7 were duplicates, 176 did not deal with oncological or PET-based radiomics, 27 articles were not human-based, 89 were technical articles, and 70 studies included less than 30 patients in the training cohort. A total of 404 articles were sought for retrieval: 5 were not written in English, 17 had no full text available and 220 studies dealt with supradiaphragmatic malignancies and were therefore excluded. Finally, 162 studies were included in the review (Figure 1). Study characteristics table is available in a separate file Table S1.

### 3.3. Quality Assessments

Mean quality score of the articles was 1.78/6, with a tendency towards constant improvement over the years (Table 1). A total of 61 (37.7%) studies included more than 100 patients each, 13 studies (8.0%) were prospectively based on acquired data, 61 (37.7%) articles described an independent validation set. The number of publications was found to be increasing each year (Table 2).

### 3.4. Gastroenteric Tract Cancers (Neuroendocrine Tumors Excluded)

#### 3.4.1. Esophageal and Gastric Cancers

Twenty-four studies on esophageal cancer were included in this review [5,6,7,8,9,10,11,12,13,14,15,16,17,18,19,20,21,22,23,24,25,26,27,28]; 1 used 18F-FDG and 18F-FLT, reporting no significant results regarding 18F-FLT [10]. The remaining 23 studies employed only 18F-FDG. The average number of patients included was 114.5 (range 30–449), with 6/24 (25.0%) studies including more than 100 patients. Moreover, 8 studies (33.3%) used an independent validation dataset and 2/24 (16.7%) were prospectively designed. Prognosis and treatment response prediction were the main investigated subjects, gathering 21/24 (87.5%) studies.

An externally validated study, conducted by Zhang et al. [19] on 190 patients, aimed at predicting lymph node metastases using pre-treatment PET radiomics of the primary tumor, achieving an AUC of 0.69 on the validation cohort. The question of overall survival prediction was raised by Foley et al. [13], however, the prognostic model developed on his cohort of 449 patients (training n = 302, internal validation n = 101, external validation n = 46) failed to be transposable to the validation groups, even after PET harmonization. Some data were oriented towards the ability of PET radiomics to predict treatment response to concurrent chemo-radiotherapy, such as in the study by Cao et al. [12], that included 159 patients with thoracic esophagus squamous cell carcinoma (AUC of 0.835 on the validation dataset).

A total of 7 studies on gastric cancer were found [29,30,31,32,33,34,35], all using 18F-FDG as radiopharmaceutical. The average number of patients included was 163.7 (range 79–214), with 5/7 (71.4%) studies including more than 100 patients, 5/7 (71.4%) using a separate validation dataset and 1/7 (14.3%) using prospective data.

Furthermore, 4 studies were conducted for diagnostic purposes: 2 for nodal involvement prediction (AUC between 0.74 and 0.81) [29,35], 1 for peritoneal involvement prediction (AUC 0.88 in the validation cohorts) [30] and 1 to differentiate between gastric cancer and primary gastric lymphoma (AUC 0.77) [31]. The remaining three were prognosis-oriented [32,33,34].

#### 3.4.2. Colorectal and anal Cancers

A total of 19 studies on colorectal cancer were included [36,37,38,39,40,41,42,43,44,45,46,47,48,49,50,51,52,53,54], all using 18F-FDG except for one [37], that employed both 18F-FLT and 18F-FDG without any reported added value on prognosis prediction. On average, 118.7 (ranging from 37 to 381) patients were included, with 7/19 (36.8%) studies including more than 100 patients, 1 using prospectively acquired data and 5/19 using a validation cohort (26.3%). Most of these studies dealt with prognosis and treatment response prediction (15/19–78.9%). The largest one, by Kang et al. [51] (training set n = 228; validation set n = 153) developed a prognosis nomogram: the radiomics signature was significantly associated with progression free survival both in training and validation sets (*p* < 0.001).

Only 1 study was conducted on patients with anal cancer (n = 189) and found that the inclusion of PET textural parameters might provide superior prediction of PFS than existing methods designed without it [55].

#### 3.4.3. Pancreatic Cancers

Pancreatic cancer was featured in 13 studies based on 18F-FDG [56,57,58,59,60,61,62,63,64,65,66,67,68]. An average of 110.7 patients was included (range: 48–198, 8 studies with more than 100 patients) with 1 (7.7%) prospective study and 4 (30.8%) using a validation cohort. A total of 8 studies (61.5%) focused on prognosis, while the remaining 5 dealt with diagnostic issues and histological characterization. Promising results were found in terms of grade of tumoral differentiation prediction [62] with a model based on a twelve-feature-combined radiomics signature that could stratify pancreatic ductal adenocarcinoma patients into grade G1 and grade G2/3 groups, with an AUC of 0.994 in the training set and 0.921 in the validation set.

#### 3.4.4. Liver Cancers

Moreover, 4 retrospective studies were identified for liver cancer [69,70,71,72] with an average of 65.5 included patients (range 47–99). Among them, 1 used a separate validation cohort [69]. Two studies focused on the response prediction of 90Y-transarterial radioembolization treatment, one using 18F-FDG [70], the other using post therapy 90Y PET [72]. One study aimed at differentiating between hepatic lymphoma and hepatocellular carcinoma (AUC 0.87 on the training set, no validation cohort) [71]. The last 1 [69] used radiomics for microvascular invasion and prognosis prediction in early-stage hepatocellular carcinoma (AUC 0.69 on the validation cohort).

### 3.5. Genitourinary Tract Cancers

#### 3.5.1. Cervical and Endometrial Cancers

A total of 22 publications on cervical cancer were retrieved; 19 of them exclusively dealt with cervical cancer [73,74,75,76,77,78,79,80,81,82,83,84,85,86,87,88,89,90,91], while the remaining 3 described multiple types of cancers, including cervical cancer [92,93,94]. All of the studies were retrospective and employed 18F-FDG. The average number of patients included in the 19 studies on cervical cancer was 105.2 (range 42–190), with 9/19 (47%) studies including more than 100 patients; 10/18 (55.6%) used dedicated validation cohorts (the remaining 1 being a validation study). Most of these studies were aimed at investigating the prognosis and disease-free survival of patients with cervical cancer. In a PET/MRI radiomics study including 102 patients with locally advanced cervical cancer (69 for the training set and 33 for the testing set), Lucia et al. [84] showed that radiomics features such as Grey Level Non-Uniformity in PET were independent prognostic factors for the outcome of patients treated with chemoradiotherapy. These findings were then successfully validated in another study using French and Canadian cohorts [77], though higher accuracy of the model was found dependent from harmonization of the radiomic features deriving from the three centers involved. In another work including 170 patients with FIGO stage IB-IVA cervical cancer, Shen et al. [76] noted that radiomics could predict pelvic or para-aortic lymph node metastases and histology.

A total of 5 studies on endometrial cancer were identified [95,96,97,98,99], all using 18F-FDG, with an average number of 121.0 (range 53–170) patients. Moreover 4 out of 5 studies (80.0%) included more than 100 patients and were validated on an independent set. No prospective studies were found. Two studies successfully used image parameters derived from the primary tumor to increase nodal staging accuracy [98,99]. Wang et al. [95] tried to use radiomics to differentiate endometrial precancerous lesions and early-stage carcinoma, however, only SUV values had high predictive diagnostic value. Finally, two articles found radiomics patterns that may orient toward underlying Lynch syndrome [96] or refine prognosis [97].

#### 3.5.2. Vulvar and Ovarian Cancers

Only preliminary studies were available in these cases, focusing on prognosis. Only one study reported applying radiomics to vulvar cancer [100]. It had a retrospective design, 40 patients included (which is not exactly a low number, vulvar cancer being part of the rare tumors family), and no validation cohort. Although the identified radiomics features did not correlate strongly with tumor biology, Moran’s I was found to predict patients’ prognosis. The only article found on advanced high-grade serous ovarian cancer [101] was retrospectively designed, it included 261 patients, and it had a separate validation set. Results from this study reported a higher prognostic performance of the investigated model combining clinical data with 18F-FDG PET radiomic features compared to other models of clinical variables alone.

#### 3.5.3. Prostate Cancer

We included 11 studies of prostate cancer radiomics [102,103,104,105,106,107,108,109,110,111,112], 8 employing 68Ga-PSMA, 2 Choline (1 18F-Fluoroethilcholine and 1 11C-Choline) and 1 using 18F-DCFPyl. An average of 71.3 patients was included (range 41–101); 2 of the studies were prospective and 4/11 studies used a validation cohort. Five studies were conducted for prognosis and treatment response prediction purposes. Interestingly, the explorative study conducted by Mazemi et al. [102] on 83 patients used a machine-learning approach on 68Ga-PSMA PET/CT to predict 177Lu-PSMA response and obtained an AUC of 80%.

#### 3.5.4. Renal Cancer

Only 1 study [113] was available on renal cancer PET radiomics and it used 18F-FDG texture analysis to predict the pathological Fuhrman nuclear grade of clear cell renal cell carcinoma. In the prospective validation cohort, the PET/CT texture parameter model had a good predictive ability, with an AUC of 0.792.

### 3.6. Neuroendocrine and Adrenal Tumors

Seven studies were found to be conducted on neuroendocrine tumors [114,115,116,117,118,119,120], half of which on pancreatic neuroendocrine tumors. The radiotracers used were 68Ga-DOTA-peptides (6/7) and 18F-FDG (1/7). Four studies aimed at predicting prognosis and four were conducted for diagnostic purposes, particularly for Ki67 prediction. Bevilacqua et al. [114] developed a model to predict grade 1 and grade 2 pancreatic neuroendocrine tumors, obtaining an AUC > 0.8.

A study [121] retrospectively performed on 49 patients with pheochromocytoma used PET textural features combined with MTV to better differentiate between sporadic and mutated tumors, and found 18F-FDG PET/CT to provide evidences for a genetic predisposition when combined with radiomics biomarkers.

### 3.7. Blood Malignancies

A total of 24 articles on lymphomas were included in this review [122,123,124,125,126,127,128,129,130,131,132,133,134,135,136,137,138,139,140,141,142,143,144,145], 13 of which studying diffuse large B-cell lymphoma (including 2 studies on gastro-intestinal lymphoma), 3 on follicular lymphoma, 3 on Hodgkin’s lymphoma, 2 on mantle cell lymphoma and 3 on other sub-types of lymphoma. 18F-FDG was the only tracer employed and all studies built radiomic models on baseline, pre-treatment PET images, often including clinical parameters and international prognostic indices. The average number of patients included was 124.7 (range 30–383), with 11/24 (45.8%) studies including more than 100 patients, 12/24 (50.0%) using a validation cohort and 3/24 (12.5%) using prospective data. Main objectives of the studies included prognosis and treatment response prediction (19/24, 79.2%) and bone marrow involvement prediction (3/24, 12.5%), with encouraging results. Among the most interesting findings, a prospective and validated study conducted by Ceriani et al. [134] on 133 patients with diffuse large B-cell lymphoma derived a radiomics score to predict progression free survival (AUC 0.706 on test data) and overall survival (AUC 0.703 on test data).

Finally, two studies used radiomics to predict bone marrow involvement in patients with suspected relapsed acute leukemia [146] and progression to symptomatic multiple myeloma [147].

### 3.8. Musculo-Skeletal and Skin Cancers

A total of 12 studies focused 18F-FDG PET radiomics on sarcomas [148,149,150,151,152,153,154,155,156,157,158,159], including 6 on osteosarcoma. On average, 72.4 patients were included (range 35–197), the higher numbers corresponding to studies including sarcomas regardless of their subtypes. Only 1 study had prospectively acquired data and 3/12 (25.0%) used validation data. Seven aimed at predicting prognosis or treatment response, 3/12 used radiomics to predict distant metastases, 2/12 were conducted for differential diagnosis purposes.

Two studies involved patients with melanomas, one of which used radiomics to differentiate pseudo progression from progression under immune checkpoint inhibition (AUC 0.82–no validation set) [160] and the other to predict BRAFV600 mutation, however, unsuccessfully [161].

### 3.9. Others

The remaining five studies included 1 study with unknown primary tumor [162], 1 study on malignant peripheral nerve sheath tumors [163] and 3 studies on liver and spinal metastases [164,165,166], with 3/5 (60.0%) prognostic studies and 2/5 (40.0%) diagnostic studies.

## 4. Discussion

### 4.1. Quality Assessment

In this work, we extracted 162 publications related to radiomics. Our composite score for the evaluation of the quality of the publications was low, estimated at 1.78/6 on average, in good agreement with previous work reporting low quality of radiomics publications [3].

Radiomics is dependent on the size of the reconstructed voxels and images post-filtering [167]. The retrospective nature of most of the available studies (>90%) and the lack of conservation of raw data prevent the performance of a standardized dedicated reconstruction protocol for radiomic purposes [2,168] and may limit the external validity of the proposed models. However, some solutions such as the ComBat harmonization method are starting to be used, with positive results [169].

The second most common obstacle to the achievement of higher quality in radiomic articles is the overfitting phenomenon. Overfitting is encountered when training is performed on too homogeneous population sets (for example, learning performed on a monocentric database with a single imaging system) or within limited data: the generated model will too closely correspond to a particular set of data and will fail to reliably predict outcomes in populations with far different characteristics [170]. Many studies have limited cohort sizes, often less than 100 patients (62.3% in our review). This low number may furthermore prevent the constitution of validation cohorts that are independent from the training base, as usually recommended [171].

Despite this, we observe an improvement in the quality of articles over the years on our composite criterion combining the number of patients, the presence of a validation cohort and the presence of prospective data.

### 4.2. Trends and Topics

The number of studies on radiomics is exponentially increasing, relying both on machine learning and deep learning approaches. In this systematic review Part 2, the most studied cancers were found to be, in order of frequency, esophageal cancer, lymphoma and cervical cancer. Most studies focused on prognostic and treatment response objectives. 18F-FDG remains the most studied tracer. Among the 21 primary tumor subtypes identified in this review, 14 were described in less than 10 publications, leaving room for future developments.

With regards to gastroenteric tumors, PET radiomics and AI analysis should be evaluated for wider application, as it has demonstrated considerable prognostic predictive validity in different settings. Interestingly, esophageal and pancreatic cancers were studied in several papers [13,65,146]: given their poor prognosis, PET radiomics and AI could offer a valid tool to personalize treatment regimens and increase precision medicine. Gastric and colo-rectal cancers could benefit from a quantitative approach both for diagnostic and prognostic purposes [34,49]. PET radiomics and AI analysis in liver cancer should be further evaluated with more specific tracers other than 18F-FDG, such as perfusion tracers and new ones such as 18F/68Ga- FAPI.

Regarding genito-urinary tumors, PET radiomics and AI analysis could help in the outcome prediction of cases with highly aggressive disease. Urinary tract cancers are less commonly studied with 18F-FDG PET/CT, even if they find its useful applicability in staging and restaging of metastatic aggressive diseases. Prostate cancer scans could be performed with different PET radiopharmaceuticals for radiomics and AI analysis purposes, such as 11C/18F-Choline and 68Ga/18F-PSMA. For PET of pelvic tumors, the interference of radioactive urine should be kept in mind in the contouring phase of radiomics and AI protocols.

Only few studies evaluated PET radiomics and AI analysis in neuroendocrine tumors [114,115,116,117,118,119,120]. In low-grade disease, 68Ga-labelled somatostatin analogues are used for staging and restaging purposes: radiomics and AI analysis could provide more information in metastatic stable disease treated with cold somatostatin analogues. In high-grade disease, the prediction of relapsing and progressive disease by 18F-FDG PET/CT could be a useful tool for personalized medicine.

Several studies evaluated the role of PET radiomics and AI analysis in blood malignancies [122,123,124,125,126,127,128,129,130,131,132,133,134,135,136,137,138,139,140,141,142,143,144,145]. Lymphoma radiomics on 18F-FDG PET/CT was more often assessed on patients with DLBCL, in retrospective cohorts and for prognostic purposes. Further studies in larger prospective cohorts and in different histotypes of lymphoma are needed. Nevertheless, other aggressive diseases such as leukemia and myeloma could take advantage of PET radiomics and AI analysis, also with different PET tracers such as aminoacidic tracers and the immuno-PET tracer 68Ga-Pentixafor.

Few studies evaluated muscolo-skeletal [148,149,150,151,152,153,154,155,156,157,158,159] and skin tumors [160,161]. These are usually aggressive neoplasias, for which new prognostic models derived by PET radiomics and AI analysis could help clinicians and patients to improve survival outcomes. Immunotherapies in metastatic melanoma could be better evaluated with new immuno-PET radiomics and AI tools, to avoid misinterpretations in stable disease as pseudo-progression due to inflammatory reactions.

### 4.3. Limitations

We applied an arbitrary threshold of 30 patients to eliminate studies that were too exposed to overfitting bias. One of the disadvantages of this selection is the potential elimination of rare pathologies from this review, as previously reported [168].

The articles were read by only one person, which exposes the risk of error in data collection. However, data collection was performed twice in order to limit this risk.

Finally, the scale used to assess the quality of the articles was practical but rather simplistic. We did not thoroughly evaluate the methodological aspects of each study. In particular, we did not check whether a satisfactory description of the factors of variability of the radiomic analyses was systematically given, namely and not exhaustively: the type of contouring used, the resampling and discretization parameters [172,173].

## 5. Conclusions

PET radiomics and AI analysis in infradiaphragmatic cancers, blood malignancies, melanoma, and musculo-skeletal cancers are an upcoming field in nuclear oncology and the number of related publications is increasing every year. Limitations encountered in the past, such as small sample size of studied populations or lack of validation cohorts, are progressively being corrected and promise further advancement towards personalized medicine.

## Figures and Tables

**Table 1:** Inclusion and exclusion criteria (PICOS systematization).

Parameter	Inclusion Criteria	Exclusion Criteria
Patients	Patients with any non-supradiaphragmatic cancer, hematological cancer, musculo-skeletal and skin cancer	Strictly supradiaphragmatic cancers
Intervention	Radiomics analysis on PET studies	
Comparator	Diagnostic performances	
Outcome	Primary outcome measures, diagnostic accuracy, area under curve	
Study design	Any trials, retrospective, prospective or concurrent cohort studies. At least 30 patients. Published in English	Reviews, expert opinions, comments, letters to editor, case reports, studies on animals or phantoms, conference reports. Less than 30 patients. Studies with no outcomes reported. Published in any language other than English

**Table 2:** Mean quality scores and number of publications per year on PET(/CT) radiomics.

Year	Quality Score (Mean-95%CI)	Number of Publications
2011	0 [-]	1
2012	-	0
2013	0.50 [0; 1.89]	2
2014	2.00 [-]	1
2015	0.75 [0; 2.63]	4
2016	1.63 [0.17; 3.08]	8
2017	1.55 [0; 3.58]	11
2018	1.76 [0; 3.79]	17
2019	1.43 [0; 3.86]	25
2020	1.79 [0; 4.13]	29
2021	1.98 [0; 4.11]	51
2022 *	2.62 [0.02; 5.21]	13 *

* Partial data.

## Data Availability

The datasets generated during the current study are available from the corresponding author on reasonable request.

## Supplementary Materials

The following are available online at https://www.mdpi.com/article/10.3390/diagnostics12061330/s1, Table S1: Study characteristics p2.xlsx’.
